# Validation and Application of an HPLC-UV Method for Routine Therapeutic Drug Monitoring of Cefiderocol

**DOI:** 10.3390/antibiotics10030242

**Published:** 2021-02-28

**Authors:** Julia Zimmer, Anka C. Röhr, Stefan Kluge, Jonas Faller, Otto R. Frey, Dominic Wichmann, Christina König

**Affiliations:** 1 Department of Pharmacy, General Hospital of Heidenheim, 89522 Heidenheim, Germany; zimmer.julia@online.de (J.Z.); Anka.Roehr@Kliniken-Heidenheim.de (A.C.R.); otto.frey@kliniken-heidenheim.de (O.R.F.); 2 Department of Intensive Care Medicine, University Medical Center Hamburg-Eppendorf, 20246 Hamburg, Germany; s.kluge@uke.de (S.K.); d.wichmann@uke.de (D.W.); 3 Department of Pharmacy, University Medical Center Hamburg-Eppendorf, 20246 Hamburg, Germany; j.faller@uke.de

**Keywords:** ESBL, Enterobacterales, cefiderocol, therapeutic drug monitoring

## Abstract

Cefiderocol is a new siderophore cephalosporin approved for the treatment of multidrug resistant bacteria including activity against carbapenem-resistant Enterobacterales and *Pseudomonas aeruginosa*. As cephalosporins are known for their high pharmacokinetic variability in critically ill patients, cefiderocol therapeutic drug monitoring might become a valuable tool. Therefore, we aimed to develop and validate a simple, rapid, cost-effective high performance liquid chromatography (HPLC) method for the quantification of cefiderocol in serum. Samples were treated for protein precipitation followed by chromatographic separation on a reverse phase column (HPLC C-18) with gradient elution of the mobile phase. Cefiderocol was detected via UV absorption and quantification was performed with the internal standard (metronidazole) method. The calibration range showed linearity from 4 to 160 mg/L. The intra and interday precision was less than 10% with a recovery rate of 81%. The method was successfully used for the analysis of subsequent serum samples of critically ill patients and showed good performance in monitoring serum levels and optimizing antibiotic therapy.

## 1. Introduction

Cefiderocol is a novel siderophore cephalosporin used for the treatment of nosocomial infections such as hospital acquired and ventilator associated pneumonia (HAP/VAP), complicated urinary tract infections (cUTIs) and difficult to treat Gram-negative bacteria in adults. As a new cephalosporin, it is closely related to both ceftazidime and cefepime. By adding a siderophore-like sidechain to the structure, drug concentration remains very high at the side of action (“Trojan horse strategy”). Its outstanding bactericidal Gram-negative spectrum shows activity against strains producing metallo-ß-lactamases and shows minimal inhibitory concentrations (MICs) of < 4 mg/L in most of the tested isolates [1]. Susceptible isolates include carbapenem resistant strains such as extended spectrum betalactamase- or carbapenemase producing Enterobacterales, *Pseudomonas aeruginosa* and *Acinetobacter baumannii* and other multidrug resistant (MDR) Gram-negative pathogens [1].

Cefiderocol is administered as a 2 g prolonged infusion (3 h) every 8 h with dose adjustments according to the renal function. Cefiderocol shows a half-life of 1.98–2.74 h and a protein binding of 58 % in healthy volunteers. It is mainly excreted unchanged via the urine and shows a volume of distribution of 13–27 L [2]. As a time dependent cephalosporin, the current dosing regimens allows one to attain a pharmacokinetic/-dynamic (PK/PD) target of 75% *f*T > MIC of 4 mg/L in most of the treated patients [2]. However, high interindividual variability in the pharmacokinetics of critically ill patients is expected. In fact, estimated cefiderocol peak concentrations (C_max_) in patients with complicated urinary tract infections ranged from 30 to 460 mg/L with 2 g of 8 hourly infused over 3 h. Additionally, drug clearance was more variable and in particular higher in patients with severe infection compared to patients without complicated urinary tract infections [3]. These results are in accordance with previous studies showing high interindividual variability in the pharmacokinetics of beta-lactam antibiotics including cephalosporins [4]. Attaining and targeting trough concentrations of beta-lactam antibiotics 4–8 times the MIC is recommended for optimal patient outcome especially in the critically ill patient cohort [5,6,7]. Additionally, beta-lactam toxicity especially of cephalosporins became more evident with reports of neurological deterioration or nephrotoxicity [8,9,10].

Currently, there is a lack of data concerning the general, potentially altered, pharmacokinetic of cefiderocol in critically ill patients, and, especially, there is no real life information on cefiderocol kinetic under extracorporeal organ support where elimination is highly variable and dependent on the mode of organ support [11,12,13]. Therefore, routine therapeutic drug monitoring (TDM) of cefiderocol would contribute to the efficacy and safety of the novel siderophore cephalosporin cefiderocol. To date, there is only a report of a complex and costly high-performance liquid chromatography (HPLC) method with mass spectrometry (MS) available for the quantification of cefiderocol [14]. Still, the implementation and use of HPLC–MS/MS techniques usually requires well trained staff and specialized laboratories and institutes, which can afford this cost- and maintenance-intensive devices. Besides the expensive device itself, chemical reagents and special reference substances, which are needed for HPLC–MS/MS analytics, are often cost drivers resulting in unaffordable fees for clinicians. In comparison, HPLC methods with ultraviolet (UV) detection are widely represented in smaller laboratories and more affordable or already in place for routine analytical purposes in hospital pharmacies. Subsequently, an HPLC-UV method would be more likely to be implemented by pharmacists or antibiotic steward-ship teams in hospital settings. Therefore, we aimed to develop a simple, rapid and cost-effective HPLC-UV method for the quantification of cefiderocol in the course of routine therapeutic drug monitoring.

## 2. Results

**Figure 1:**
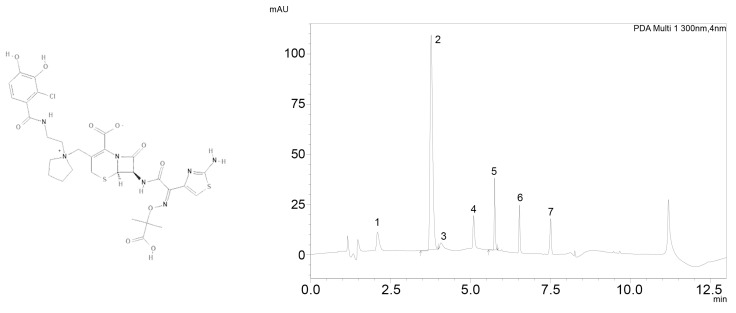
Left: chemical structure of cefiderocol [15]; right: chromatogramm showing 1:cefepime; 2: metronidazole (internal standard); 3:meropenem; 4: ceftazidime; 5: cefiderocol; 6: cefotaxime; 7: cefuroxime.

The tested and validated HPLC-UV method allows easy detection of cefiderocol (see Figure 1) in spiked serum and in patient serum samples. Sufficient peak separation from other beta-lactam antibiotics quantifyable with this method was achieved with the given HPLC-UV parameters (see Figure 1 right panel).

Cefiderocol peaks could be identified at a retention-time of 5.5 min at a wavelength of 300 nm showing proper shape and intensity. The method was in line with the Valistat 2.0 (ARVECON GmbH, Walldorf Germany) validation criteria as required by the German Society of Toxicology and Forensic Chemistry (GTFCh) and used to analyze clinical samples from patients treated with cefiderocol [16]. Calibration curves were linear over the concentration range of 4–160 mg/L with a correlation coefficient of 0.999. The bias for accuracy within the samples analyzed on different days was below the acceptance criteria of ±15% along the concentration range. The lower limit of quantification was determined to be 4 mg/L with a maximum relative standard deviation (RSD) of ±20%. The acceptance criteria of ±15% and ±20% at the lower limit of quantification were applied and fulfilled for precision and for inter- and intraday assay performance (see Table 1). Inter- and intraday precision showed a low overall RSD of 3.3–4% indicating good assay performance. Within 110 intensive care patients not receiving cefiderocol there was no interference of other drugs at the given retention time and wavelength.

**Figure 2:**
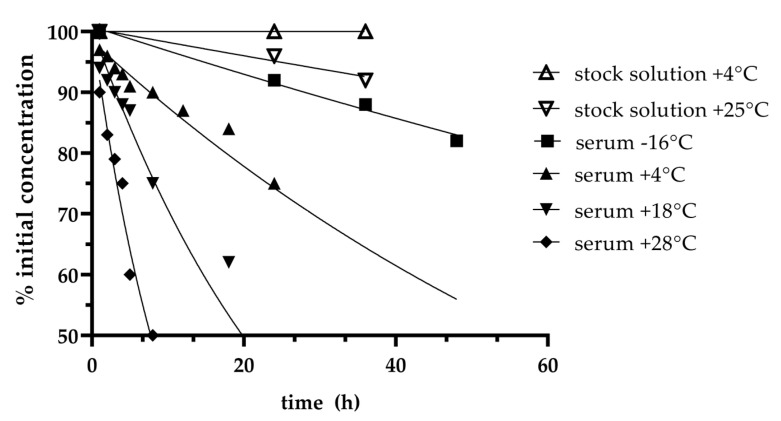
Cefiderocol stability over time.

Stability of spiked samples and patient samples dependent on the storage conditions was low (see Figure 2). At room temperature samples were stable for up to 2 h. When stored refrigerated (2–8 °C) stability was up to 8 hours, whilst stored frozen at −20 °C up to one day and at −80 °C for at least 31 days (see Figure 2). Stability of extracted samples in the auto sampler at 10 °C was within a ±10% range for at least 24 h. Aqueous stock solutions were stable for 24 h at room temperature and for 6 months at −80 °C (data not shown).

Patient samples of three critically ill patients receiving cefiderocol for infection with Gram-negative bacteria were drawn for therapeutic drug monitoring purposes. The samples were subsequently processed and analyzed according to the described method. If rapid transport (within 1–2 h) to the laboratory could not be guaranteed, patient samples were centrifuged at 8.000× *g* for 3 min. The resulting supernatant was immediately frozen and stored at −80 °C until further analysis. For transport, samples were placed on dry ice to maintain low temperatures. The measured cefiderocol concentrations in serum and patient characteristics are reported in Table 2. No interference with other drugs used in these patients was observed. Moreover, peak purity was monitored and continuously existent when analyzing cefiderocol in the patient samples.

Table 2 reports three critically ill patients treated with cefiderocol for hospital acquired pneumonia (HAP) due to a multidrug resistant *Pseudomonas aeruginosa* and *Acinetobacter baumannii.* The serum cefiderocol concentrations were measured before the next infusion (trough levels) was due. The patient’s estimated glomerular filtration rate (eGFR) was calculated by the chronic kidney disease epidemiology collaboration equation (CKD-EPI) [17] or neglected if continuous renal replacement therapy (CRRT) was present. The cefiderocol dosage regimen of patient one was subsequently adapted according to the reported trough concentrations of 70 mg/L and with respect to an ongoing deterioration of renal function (CKD-EPI eGFR of 22 mL/min/1.73 m^2^). Even though the dose was reduced, patient one maintained high concentrations of 49 mg/L by the end of the dosing interval. Patient two and three with severe septic shock and continuous renal replacement therapy showed a trough concentration of 12 and 18 mg/L at the first day of therapy. Unfortunately, patient two and three died due to ongoing deterioration of multi organ failure.

## 3. Discussion

Here, we reported a simple HPLC-UV “bedside” method for the quantification of cefiderocol in human serum. Compared to more sophisticated HPLC methods with mass spectrometry (MS), this method allows a cost-effective analysis of clinical specimens. Even though, a previously reported HPLC–MS/MS method showed a lower limit of quantification of 0.1 mg/L, aiming for quantifications in the forensic scale are not necessary since therapeutic drug monitoring is the major application of this method [14]. Our method provides high sensitivity for clinically observed drug concentrations, as clinical studies reported trough concentrations of > 9 mg/L [18]. Moreover the lower limit of quantification of 4 mg/L of the current method is below the reported trough concentrations and covers the MIC of 2 mg/L for susceptible Gram-negative bacteria [19]. Additionally, the levels of the quality controls (160 and 31 mg/L) were chosen to cover a broad concentration range of 5–123 mg/L observed in clinical safety studies in healthy volunteers [18].

As Saisho and Miyazaki et al. reported the amount of cefiderocol metabolites being less than 10%, further evaluation of metabolites was dispensed [18,20]. Additionally, metabolite occurrence showed no major difference when evaluating different dosing regimens (single vs. multiple dosing), indicating that metabolite accumulation is unlikely [18,20]. As the reported HPLC-UV method is already implemented for the quantification of beta-lactam antibiotics (e.g., meropenem or piperacillin) in critically ill patients, multiple chromatograms of intensive care patients showed no interference at the given retention time and wavelength of cefiderocol.

Choosing conventional metronidazole for the internal standard, allowed a reasonable, available and easy accessible substance to monitor assay performance. Since cefiderocol is used for specific rather than empirical treatment, there is no need for routine combination with anaerobic anti-infectives such as metronidazole. Therefore, misinterpretation of chromatograms by false high internal standard peaks is negligible. Additionally, as it is advisable that antibiotic stewardship teams supervise serious infections treated with reserve antibiotics such as cefiderocol, there is usually a broad transparency of concurrent drug therapy [21,22].

The stability studies showed low cefiderocol stability in spiked sheep and patient serum with a stability of only 8 h when the samples are stored refrigerated at 2–8 °C. This finding was surprising, since the manufacturer states a stability of 24 h when the reconstituted infusion is stored refrigerated with protection from light. Moreover, studies with radiolabeled cefiderocol showed only minor degradation/metabolism in vivo [20]. Besides, other beta-lactams such as piperacillin, meropenem and ceftazidime show low stability in plasma and whole blood too [23]. Future studies are needed to identify the underlying mechanism of degradation for cefiderocol. Long-term stability was quite poor, with samples remaining stable for at least 31 days at −80 °C. Based on the intention to establish a therapeutic drug monitoring method for cefiderocol with same-day reporting even a stability of 8 h (2–8 °C) is sufficient when sample preparation and quantification will be performed in house. Samples should be stored and transported at least at −20 °C if transfer to external laboratories is needed. If sample processing is delayed for more than 20 h storage conditions should be set to −80 °C. 

As a time-dependent antibiotic it is crucial for a clinical outcome to maintain cefiderocol concentrations above the MIC by the end of the dosing interval [24,25,26]. According to European Society of Antimicrobial Susceptibility Testing (EUCAST), the current susceptibility breakpoint for cefiderocol for Enterobacterales and *Pseudomonas aeruginosa* is defined as 2 mg/L [19]. When targeting for trough concentrations MIC and the protein binding of cefiderocol has to be taken into account. Assuming a protein binding of approximately 50% a total cefiderocol trough concentration of 4 mg/L (equals a free fraction of approximately 2 mg/L) would be adequate. To attain higher targets (4 × MIC) a trough level of 16 mg/L would be sufficient. When analyzing specimens from three critically ill patients we observed serum concentrations remaining well within the concentrations of the calibration range. Patient one showed an initially high trough level of 70 mg/L representing a free fraction of approximately 35 mg/L, which equals 17 × MIC of 2 mg/L. In clinical trials total trough levels ranged between 9.66 and 13 mg/L in healthy volunteers with trough concentrations above 50 mg/L in patients with renal impairment [14]. Even though the dose was reduced, the patient maintained trough concentrations (total 49 mg/L; approx. free fraction 25 mg/L) above 10 × MIC of 2 mg/L by the end of the dosing interval. Within this critically ill patient, the initial eGFR value of 67 mL/min/1.73 m^2^ was not reliable to guide cefiderocol dosing. This is in concordance with Udy et al. and Baptista et al. who showed a significant discrepancy of CKD-EPI eGFR and measured creatinine clearances in the critically ill patient cohort [27,28]. The measured total trough concentration (12 mg/L) of patient two corresponds to a free fraction of 6 mg/L, which represents at least 3 × MIC of 2 mg/L by the end of the dosing interval. This value is in concordance with the range reported in clinical trials [18] but still unexpected since the patient was treated with CRRT for acute renal impairment. A third patient with a similar severity of disease and CRRT supports this finding by showing a cefiderocol trough concentration of 18 mg/L. These findings again, confirm the existence and moreover the extent of variability in drug elimination especially in the critically ill patient cohort, which is of major importance to optimize antibiotic therapy [4]. Due to the novelty and antimicrobial activity of cefiderocol its use is currently exceptional and only three patients were eligible for therapeutic drug monitoring. Moreover, since previous studies could prove potential benefit of therapeutic drug monitoring of beta-lactam and other antibiotics on good clinical outcome, measurement of cefiderocol might become a valuable tool [29,30]. Additionally, the European Society of Intensive Care Medicines recommends therapeutic drug monitoring for beta-lactam antibiotics as standard of care despite the knowledge of the lack of routinely available quantification methods [31]. In laboratories where therapeutic drug monitoring of beta-lactam antibiotics is performed frequently an integration of cefiderocol in existing HPLC methods is a reasonable way to minimize costs and effort. Especially, for novel substances such as cefiderocol, which is currently used only in selected cases rather than empirically. This approach also allows for short turn-around-times, which is crucial for patients where dose-optimization might influence survival such as in a severe infection.

Therefore, this routine and easy to handle HPLC-UV method for bedside therapeutic drug monitoring of cefiderocol is of particular importance for closing the gap towards precision dosing in critically ill patients [24,32].

## 4. Materials and Methods

Cefiderocol (Fetroja^®^) was obtained from Shionogi (Osaka, Japan) as a regular vial containing powder (1 g per vial) for reconstitution. One vial Fetroja^®^ contains cefiderocolhemisulfattosilat, which is equivalent to 1 g of cefiderocol. The powder was weighted and dissolved with water for injection (Ampuwa, Fresenius Kabi, Germany) to prepare the stock solution with a concentration of 1000 mg/L. To prepare calibration standards (20 mg/L) and quality controls (31 and 160 mg/L) sheep serum (Fiebig Nährstofftechnik, Germany) was spiked directly with aliquots of the stock solution. The calibration standards and quality controls were immediately frozen at −80 °C, replaced every two weeks and thawed just before use. Protein precipitation was performed by adding 200 µL of an acetonitrile/methanol (1:1) mixture of HPLC grade to 100 µL patient serum. The protein precipitation mixture contained 100 mg/L metronidazole (metronidazole 5 mg/mL, B.Braun, Melsungen, Germany) as an internal standard. Subsequently, samples were mixed for 10 s and centrifuged at 8000 ×*g* for 3 min. Next, 100 µL of the resulting supernatant was further diluted with 500 µL of solvent A containing 0.1% formic acid in water of HPLC grade.

**Figure 3:**
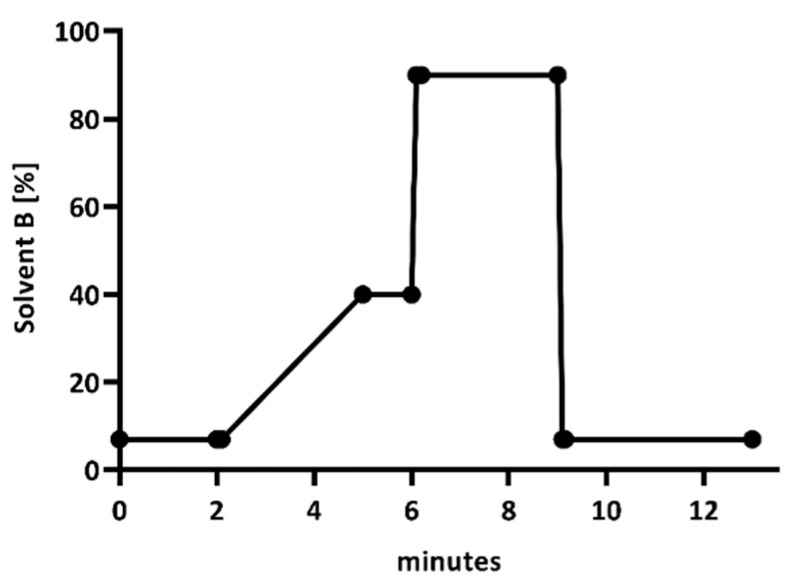
HPLC gradient of solvent B (0.1% formic acid in acetonitrile).

An aliquot of 10 µL was injected onto the HPLC-UV system equipped with a diode array detector (Nexera-I 3D plus, Shimadzu; Duisburg, Germany). Chromatographic analysis was performed using a reverse phase column Shim-pack XR-ODS III with 2.2 µm particle size (150 mm × 2 mm, Shimadzu, Duisburg, Germany) in combination with a column guard (Shim-pack GISS-HP (G) C18; 3 µm; 10 mm × 2.1 mm, Shimadzu, Duisburg, Germany). Separation was performed with using a gradient of solvent B containing of 0.1% formic acid in acetonitrile (HPLC grade) with a flow-rate of 0.35 mL/min (see Figure 3).

**Figure 4:**
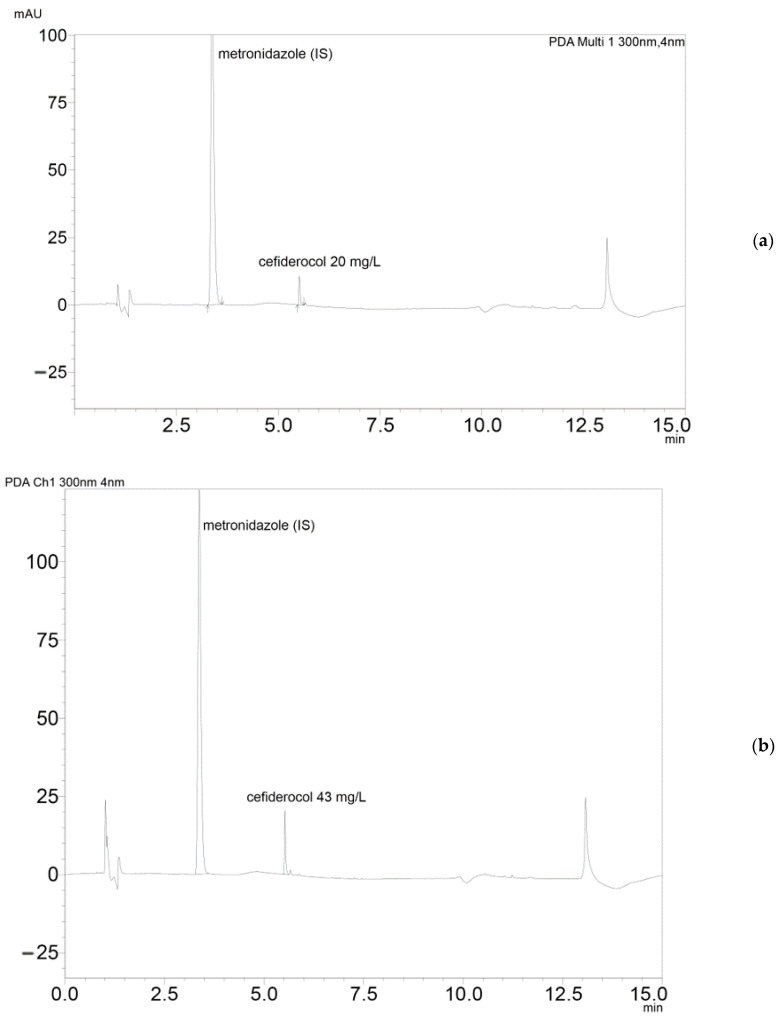
(**a**) Chromatogram of cefiderocol calibration standard concentration (20 mg/L) and (**b**) chromatogram of cefiderocol in patient serum.

The auto sampler and column were tempered to 10 °C and 45 °C, respectively. Cefiderocol was monitored at a wavelength of 300 nm showing good UV-absorption (Figure 4 showing chromatograms) with a retention time of 5.5 min.

### Assay Validation

For peak identification aqueous solutions of cefiderocol (20 mg/L) were analyzed with the above mentioned method and assessed for intensity (area), shape and retention time. Further samples and validation was performed with spiked sheep serum.

Linearity was conducted via a calibration curve with seven samples ranging from 4 to 160 mg/L. Each of the samples was analyzed six times and evaluated by peak area vs. target concentration with an accepted correlation coefficient of >0.95.

In order to determine assay precision, the relative standard deviation (RSD, reported in %) of the results of six samples of a low (31 mg/L) and high concentration (160 mg/L) was calculated. Differentiation between inter- and intraday precision was performed additionally, with intraday precision being performed on eight consecutive days. A general RSD of ±15% and ±20% at the lower limit of quantification was accepted.

Accuracy was evaluated by analyzing two samples of two different concentrations on eight different days. The degree of accuracy was determined by the bias, with ±15% and ±20% at the lower limit of quantification being acceptable.

Interference with other drugs was assessed by cross-examination of 110 serum specimens of patients not receiving cefiderocol but other anti-infectives and other intensive care specific drugs. The chromatograms were examined for interfering peaks at the retention time (5.5 min) and detection wavelength of 300 nm for cefiderocol. Additionally, peak purity and component identification was determined by recording a complete UV spectrum of the peak facilitated by a diode array detector at the retention time of 5.5 min.

Stability of cefiderocol was studied at different conditions including room temperature (18, 25 and 28 °C), refrigerated at 2–8 °C and frozen at −16 °C and −80 °C. Stability was tested for a pool of spiked serum and patient serum samples for two months. Moreover, aqueous stock solutions were tested at room temperature, refrigerated at 2–8 °C and −80 °C. For stability testing two samples were thawed out and analyzed subsequently. Sample stability was defined by the timeframe where samples remained a concentration > 90% of their baseline concentration. Additionally, stability of extracted samples in the auto sampler at 10 °C during processing time was checked for a day shift of a maximum of 24 h.

## 5. Conclusions

With this HPLC-UV method, cefiderocol therapeutic drug monitoring can be performed on a daily basis with a quick turn-around-time based on a cost-effective analytical method, which can be easily implemented in smaller laboratories or hospital pharmacies. This method showed good performance, precision and accuracy in clinical patient specimens. When transferring cefiderocol monitoring in clinical practice as we did, resulted in highly variable drug exposure in need for dose adaptions. Without therapeutic drug monitoring dose adaptions of antimicrobials especially in the critically ill patient cohort often remain a black box to the physician. Therefore, cefiderocol therapeutic drug monitoring enables clinicians to monitor antibiotic therapy and to perform real-time dose adjustments based on trustworthy drug values. Nevertheless, further studies are needed to investigate the optimal antibiotic exposure of beta-lactam antibiotics and cefiderocol to define the therapeutic range for safety and efficacy.

## Figures and Tables

**Table 1:** Results of the HPLC-UV validation for cefiderocol in serum.

	Low Concentration (32 mg/L)	High Concentration (160 mg/L)
Intraday precision (RSD) (%)	3.3	3.8
Interday precision (RSD) (%)	4.0	4.0
Accuracy (Bias) (%)	−5.3	8.2
Stability		
20 °C (h)	2	
2–8 °C (h)	8	
−20 °C (h)	28	
−80 °C (days)	31	

h = hours, RSD= relative standard deviation.

**Table 2:** Patient characteristics and cefiderocol measurements.

Patient	Age (y)	Weight (kg)	eGFR×n	Dose (mg/d)	C_min_ (mg/L)	Infection	Pathogen
1	69	60	67	3000	70	HAP	*P. aeruginosa*
			22	2000	49		
2	75	69	CRRT	6000	12	HAP, sepsis	*A. baumannii*
3	58	85	CRRT	6000	18	HAP, sepsis	*A. baumannii*

C_min_ = trough level; CRRT = continuous renal replacement therapy; eGFR = estimated Glomerular filtration rate in mL/min/1.73 m^2^; HAP—hospital acquired pneumonia.

## Data Availability

No new data were created or analyzed in this study. Data sharing is not applicable to this article.
